# Supplementary material for: N-glycosylation is a potent regulator of prion protein neurotoxicity
Source: J Biol Chem. 2023 Jul 27;299(9):105101. doi: 10.1016/j.jbc.2023.105101 (PMC10469999; doi:10.1016/j.jbc.2023.105101)
Supplement: Supporting information [file mmc1.pdf]

N-Glycosylation is a Potent Regulator of Prion Protein Neurotoxicity

Kevin M. Schilling[a], Pooja Jorwal[b], Natalia C. Ubilla-Rodriguez[a], Tufa E. Assafa[a], Jean R. P. Gatdula[b], Janelle Vultaggio[b], David A. Harris*[b], and Glenn L. Millhauser*[a]

[a] 1156 High Street. Department of Chemistry and Biochemistry, University of California, Santa Cruz, CA, 95064, United States

[b] 72 E. Concord St. Department of Biochemistry, Boston University, Chobanian & Avedisian School of Medicine, Boston, MA, 02118, United States

*Correspondence: DA Harris: daharris@bu.edu, or GL Millhauser: glennm@ucsc.edu

+1 617-358-4280 (phone)	+1 831 459 2176 (phone)
+1 617-358-4353 (fax)	+1 831 459 2935 (fax)

Supporting Information

Figure S1: Representative traces from whole cell patch clamp recordings.

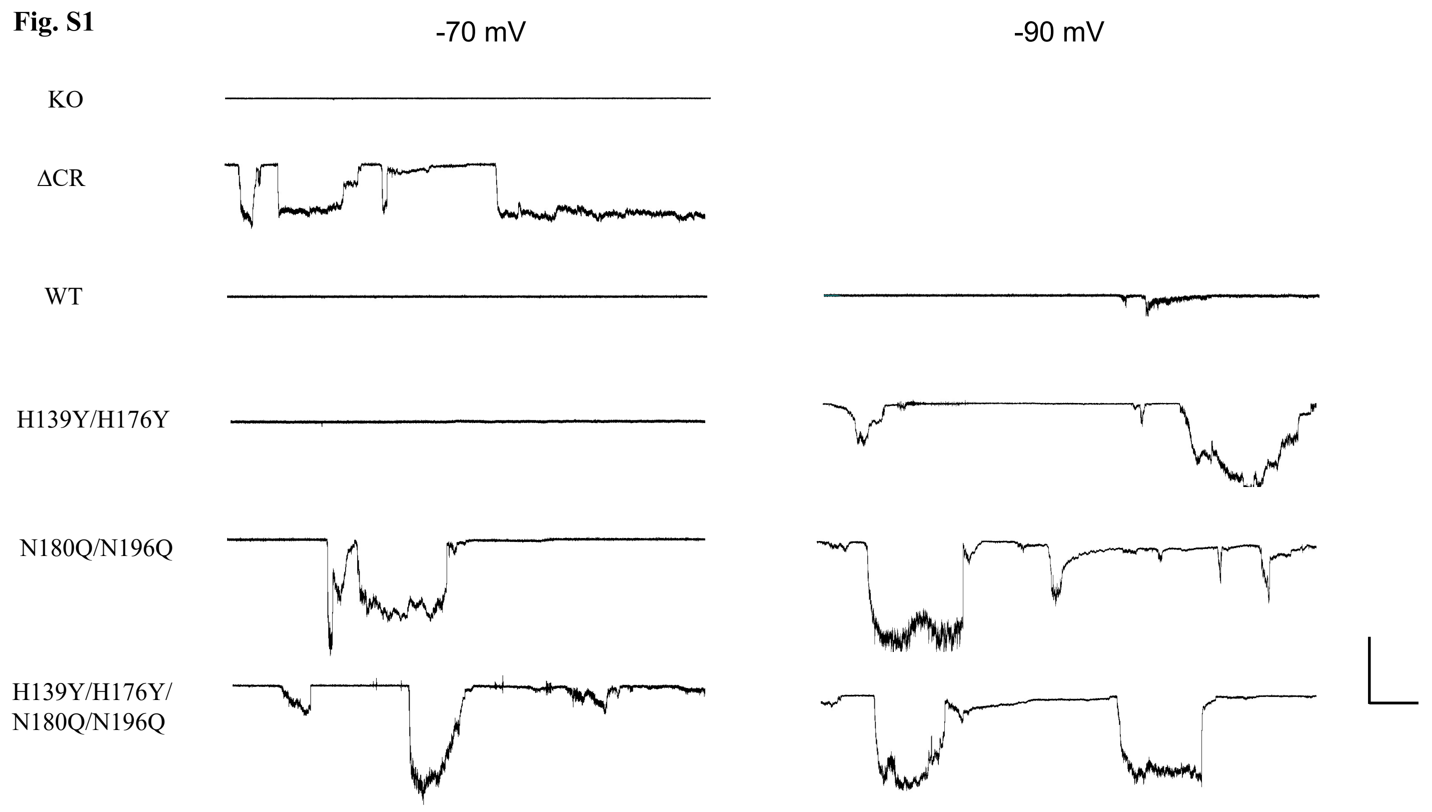

Figure S1: Representative traces of currents recorded from either untransfected N2a cells (KO) or N2a cells expressing following forms of PrP : ΔCR, WT, H139Y/H176Y, N180Q/N196Q and H139Y/H176Y/N180Q/N196Q. Quantification of the currents is shown in figure 7. Holding potential was either -70 mV (left) or -90 mV (right). Scale bars: 1 nA, 30 s.
